# Supplementary material for: HuR (ELAVL1) Stabilizes SOX9 mRNA and Promotes Migration and Invasion in Breast Cancer Cells
Source: Cancers (Basel). 2024 Jan 16;16(2):384. doi: 10.3390/cancers16020384 (PMC10813878; doi:10.3390/cancers16020384)
Supplement: Supplementary file 1 [file cancers-16-00384-s001.zip › cancers-2720498-supplementary.pdf]

Supplementary Materials

HuR (ELAVL1) Stabilizes *SOX9* mRNA and Promotes Migration and Invasion in Breast Cancer Cells

Supplementary Datasets

Dataset S1. RNA seq analysis of the gene expression profile of shHuR-1 silenced cells versus shSCR control cells.

Dataset S2. HuR candidate targets selection.

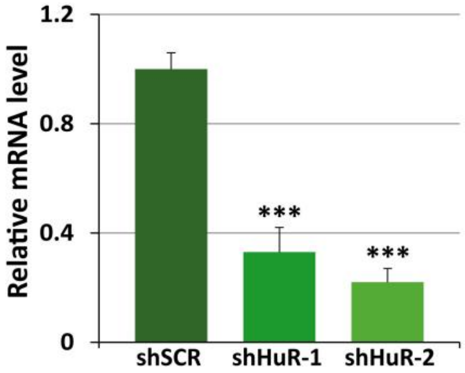

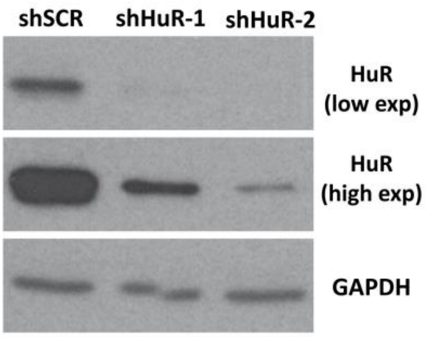

Figure S1. Silencing of HuR in Hs578T cell line. A) HuR silencing using two specific shRNAs. (shHuR-1 and shHuR-2) and a scramble control (shSCR) sequence was confirmed by qPCR. Data represent the mean ± SEM of three independent experiments with triplicate assays. The p-value was calculated using a two-sided unpaired Student's t-test. (***p < 0.001). B) Western blot analysis of HuR levels in control (shSCR) or HuR-silenced (shHuR-1, shHuR-2) cells. GAPDH was used as a loading control.

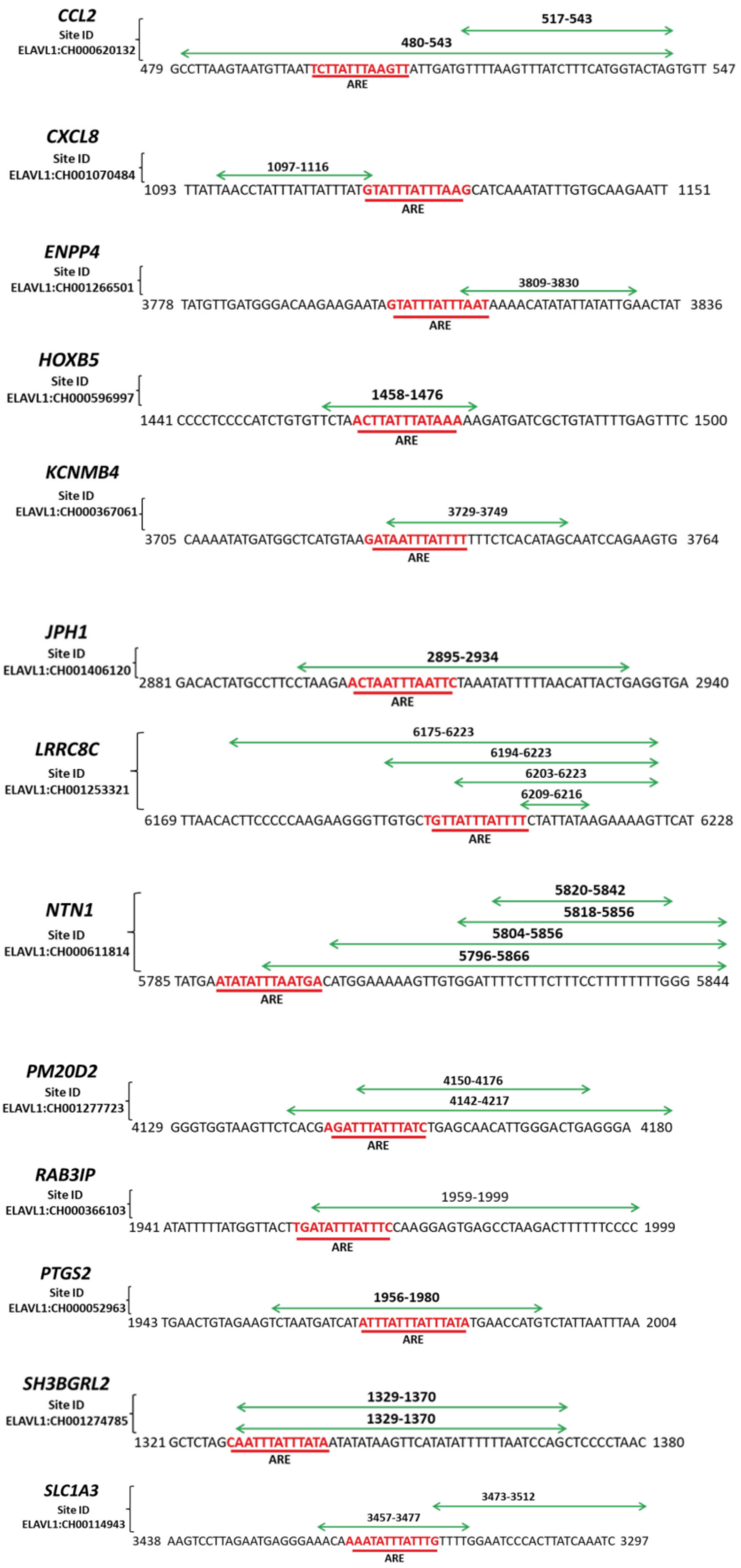

CCL2
Site ID
ELAVL1:CH000620132
517-543
480-543
479 GCCTTAAGTAATGTTAATTCTTATTTAAGTTATTGATGTTTTAAGTTTATCTTTCATGGTACTAGTGTT 547
ARE
CXCL8
Site ID
ELAVL1:CH001070484
1097-1116
1093 TTATTAACCTATTTATTATTTATGTATTTATTTAAGCATCAAATATTTGTGCAAGAATT 1151
ARE
ENPP4
Site ID
ELAVL1:CH001266501
3809-3830
3778 TATGTTGATGGGACAAGAAGAATAGTATTTATTTAATAAAACATATATTATATTGAACTAT 3836
ARE
HOXB5
Site ID
ELAVL1:CH000596997
1458-1476
1441 CCCCTCCCCATCTGTGTTCTAACTTATTTATAAAAAGATGATCGCTGTATTTTGAGTTTC 1500
ARE
KCNMB4
Site ID
ELAVL1:CH000367061
3729-3749
3705 CAAAATATGATGGCTCATGTAAGATAATTTATTTTTTCTCACATAGCAATCCAGAAGTG 3764
ARE
JPH1
Site ID
ELAVL1:CH001406120
2895-2934
2881 GACACTATGCCTTCCTAAGAACTAATTTAATTCTAAATATTTTTAACATTACTGAGGTGA 2940
ARE
LRRC8C
Site ID
ELAVL1:CH001253321
6175-6223
6194-6223
6203-6223
6209-6216
6169 TTAACACTTCCCCCAAGAAGGGTTGTGCTGTTATTTATTTTCTATTATAAGAAAAGTTCAT 6228
ARE
NTN1
Site ID
ELAVL1:CH000611814
5820-5842
5818-5856
5804-5856
5796-5866
5785 TATGAATATATTTAATGACATGGAAAAAGTTGTGGATTTTCTTTCTTTCCTTTTTTTTTGGG 5844
ARE
PM20D2
Site ID
ELAVL1:CH001277723
4150-4176
4142-4217
4129 GGGTGGTAAGTTCTCACGAGATTTATTTATCTGAGCAACATTGGGACTGAGGGA 4180
ARE
RAB3IP
Site ID
ELAVL1:CH000366103
1959-1999
1941 ATATTTTTATGGTTACTTGATATTTATTTCCAAGGAGTGAGCCTAAGACTTTTTTCCCC 1999
ARE
PTGS2
Site ID
ELAVL1:CH000052963
1956-1980
1943 TGAACTGTAGAAGTCTAATGATCATATTTATTTATTTATATGAACCATGTCTATTAATTTAA 2004
ARE
SH3BGRL2
Site ID
ELAVL1:CH001274785
1329-1370
1329-1370
1321 GCTCTAGCAATTTATTTATAATATATAAGTTCATATATTTTTTAATCCAGCTCCCCTAAC 1380
ARE
SLC1A3
Site ID
ELAVL1:CH00114943
3473-3512
3457-3477
3438 AAGTCCTTAGAATGAGGGAAACAAAATATTTATTTGTTTTGGAATCCCACTTATCAAATC 3297
ARE

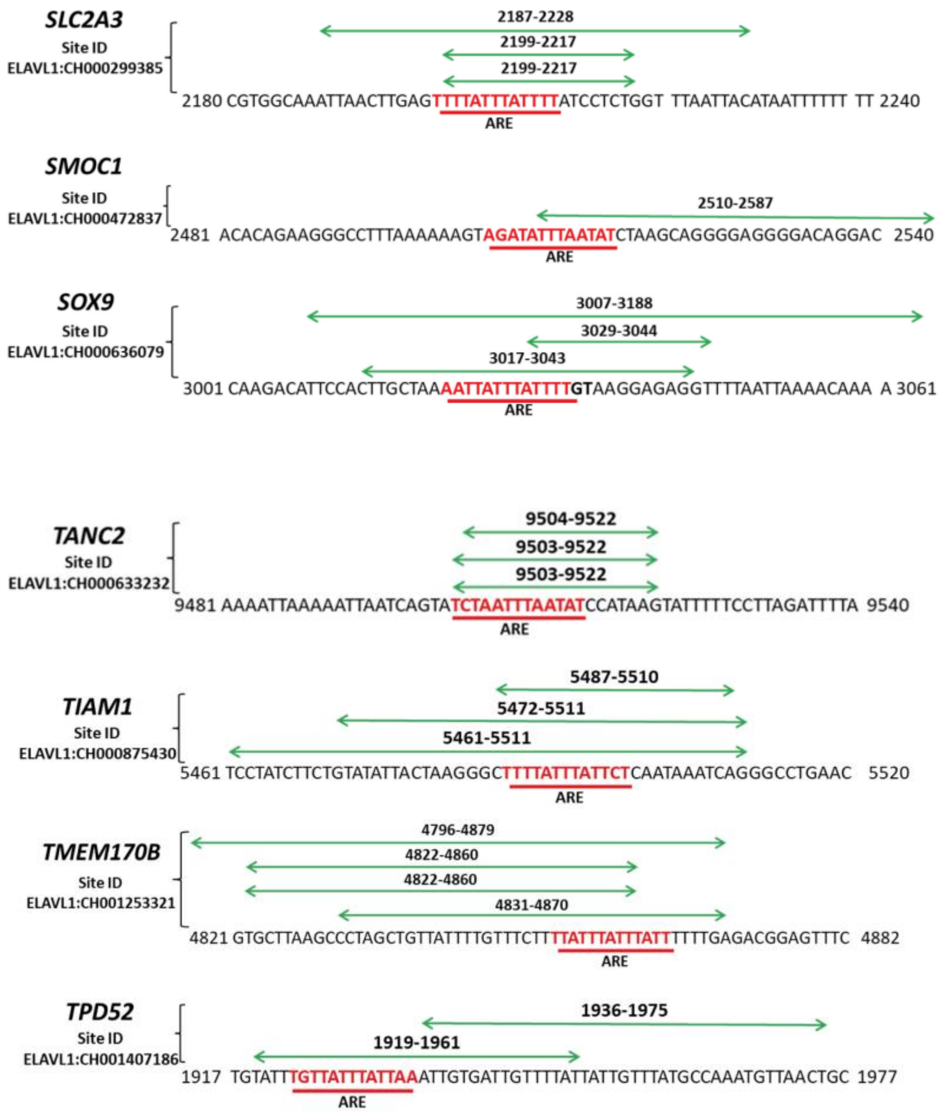

Figure S2. Location of the ARE and HuR binding sites in the 3'UTR of CCL2, CXCL8, ENNP4, HOXB5, KCNMB4, JPH1, LRRC8C, NTN1, PM20D2, RAB3IP, PTGS2, SH3BGRL2, SLC1A3, SLC2A3, SMOC1, SOX9, TANC2, TIAM1, TMEM170B, and TPD52 genes. ARE sequence in the 3' UTR is highlighted in red. Green arrows denote the coordinate of the HuR binding sites as obtained from ENCORI Site ID indicated on the left.

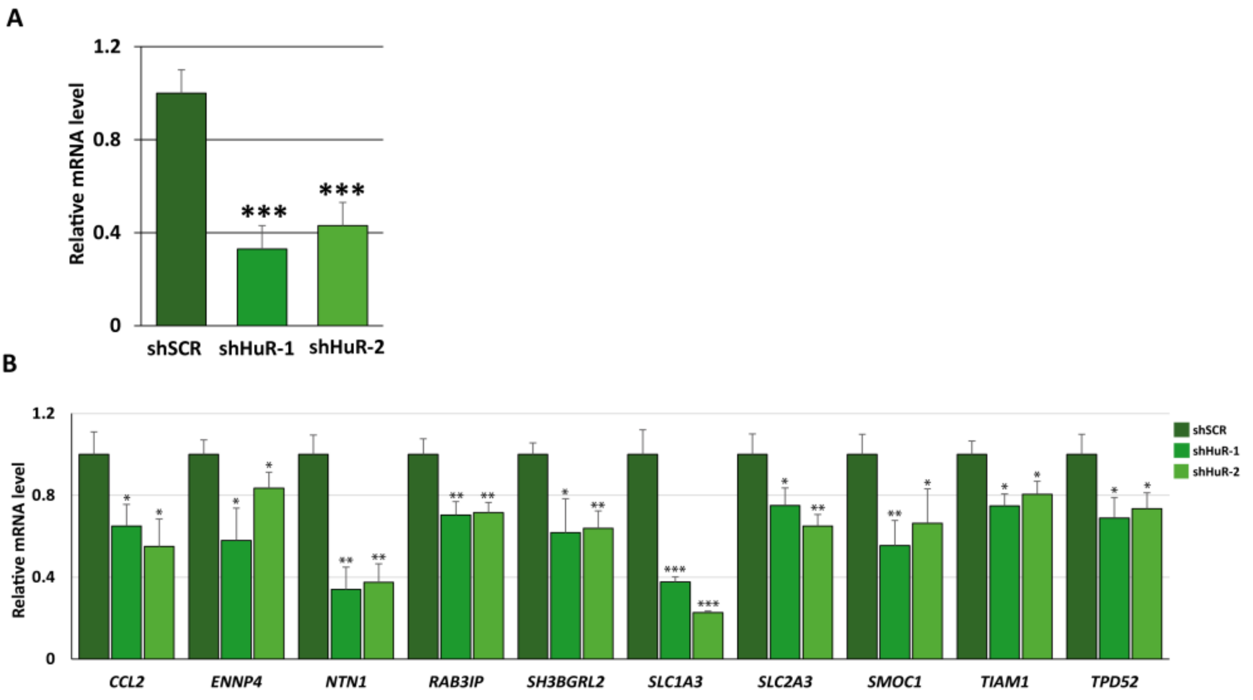

Figure S3. Validation of downregulation of putative targets in HuR-silenced BT549 cells by quantitative RT-PCR. A) Silencing of HuR in BT549 cells using two specific shRNAs (shHuR-1 and shHuR-2) and a scramble control (shSCR) was confirmed by qPCR. B) Validation of downregulation of putative targets in BT549 HuR-silenced cells by quantitative RT-PCR. Data represent the mean ± SEM of three independent experiments with triplicate assays. The p-value was calculated using a two-sided unpaired Student's t-test. (*$p < 0.05$; **$p < 0.01$; ***$p < 0.001$).

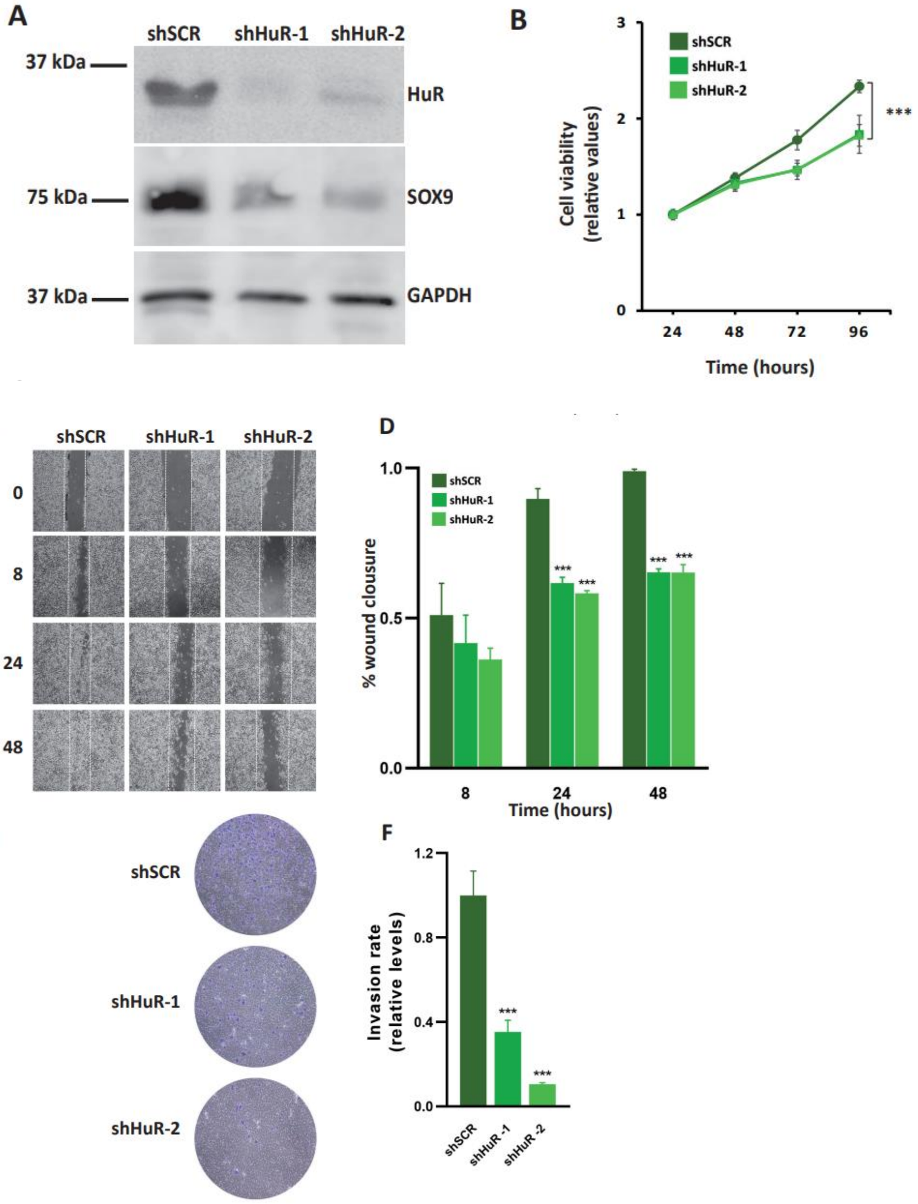

Figure S4. HuR silencing diminishes proliferation, migration, and invasion in BT549 cells. (A) Western blot analysis of HuR and SOX9 levels in control (shSCR) or HuR-silenced (shHuR-1, Scheme 2.) cells. GAPDH was used as a loading control. (B) Cell viability analyses of control (shSCR) or HuR-silenced (shHuR-1, shHuR-2) cells. MTT assays were performed at the indicated time points after seeding. Data were normalized to the value at 24 hours. Error bars represent the mean ± SEM of three independent experiments assayed in quintuplicate. (C) Cell motility of the indicated clones was analyzed by wound healing assay. Images were taken at 0, 8, 24, and 48 hours (h) after culture scratch (left panel) and area closure was quantified using ImageJ (right panel). Results represent the mean ± SEM of three independent experiments. (D) Cells were seeded in the upper chambers of Transwell, allowed to migrate for 18 h, and photographed. Left: representative images of the lower chamber (invading cells). Right: percentage of invasiveness by direct measurement with ImageJ. Values represent mean ± SEM. from three independent experiments performed in triplicate. (***$p < 0.001$).

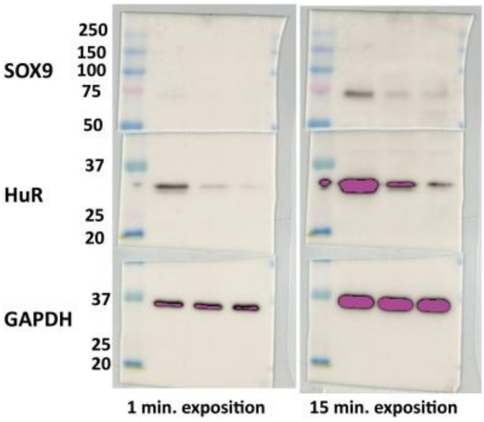

Figure S5. Uncropped Western blot images from Figure 3C.

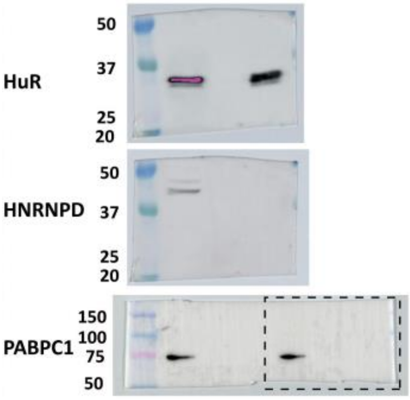

Figure S6. Uncropped Western blot images from Figure 4.

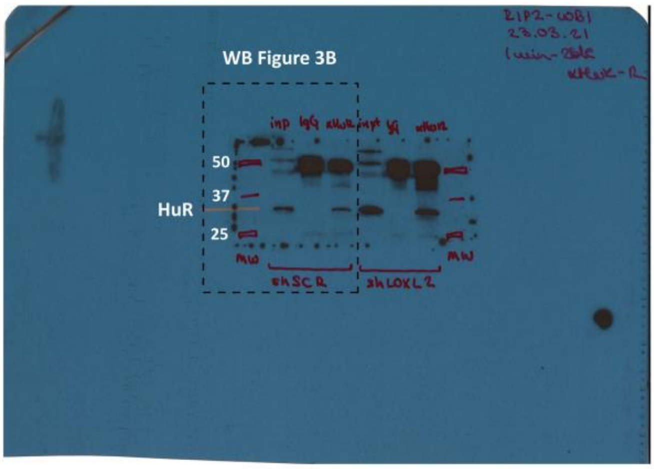

Figure S7. Uncropped Western blot images from Figure 3B.

Table S1. List of primers used for qPCR analysis.

Gene	Forward primer (5'→3')	Reverse primer (5'→3')
ACTB	TTGTTACAGGAAGTCCCTTGCC	ATGCTATCACCTCCCCTGTGTG
AKR1C2	CAGAGGTTCCTAAAAGTAAAGC	TGTGCAGAATCAATATGGTG
CCL2	AGACTAACCCAGAAACATCC	ATTGATTGCATCCTGGCTG
CLDN1	TTGGCATGAAGTGTATGAAG	ACCTGCAAGAAGAAATATCG
ELAVL1	GATCAGACTACAGGTTTGTC	TTGAAACTGGGTAATTGCCTC
ENPP2	ATAAAACAGTACGTGGAAGG	CTACCCATTTTGATTCGTCC
ENPP4	CCTGCATCGATCATTCATAC	CATTCATATGAGGGCTACAG
FAM59A	CATCGTCACTAAAACTGACAC	ATAATGGTTAGGCCATGAGAG
GAPDH	CTTCACCACCATGGAGGAGGC	GGCATGGACTGTGGTCATGAG
ID1	ACTAAGTCACCAGAGACTTTAG	AAATCTGAGAAGCACCAAAC
ITGA2	GGTGGGGTTAATTCAGTATG	ATATTGGGATGTCTGGGATG
LAMA3	GAATCAGTTGCTCAACTACC	TCAGTCAGTTCTCTTTCCAG
NTN1	CTGCATAAAGATCCCTGTAG	ATGTTAATCTTCAGCTTCCC
PTX3	AGAGAGAGTTGAGACCAATC	AAACAATTGTCCCCTCTGTTC
RAB27B	GAACTGGCTGACAAATATGG	GTTTCTACAGCTTTCTCCAC
RAB3IP	GTTGATCAGATGTTTTGGGAG	CCTCTTTGAAATAACCCAGC
RNA18S	CGAACGTCTGCCCTATCAACTT	ACCCGTGGTCACCATGGTA
SH3BGRL2	AAGGCAGAACCTTAGAGAAG	CACAGTGATGCATTAGGTAAG
SLC1A3	CCCTTACAAAATCAGAAAATTG	TTTTCTAGGAGGGTCTCTTC
SLC2A3	GGATAACTATAATGGGATGAGC	CCACAATAAACCAGGGAATG
SMOC1	ATACTTACACTGGGTACTGC	TGAACCTGAACATACAGGAG
SOD2	ATCATACCCTAATGATCCCAG	AGGACCTTATAGGGTTTTCAG
SOX9	CTCTGGAGACTTCTGAACG	AGATGTGCGTCTGCTC
TIAM1	AAGGATGCACGCTATTTTTC	GAAGACAAAGTCCTTCTTGG
TPD52	AGGTAGAAGAAGAAATCCAGAC	CCTGTAGAGAATTGATTCCAAG

Table S2. RNA sequence used in RNA pulldown experiments.

RNA	Sequence (5'→3')
AREwt	GCUAAAAUUAUUUAUUUUGUAAGG
AREmut	GCUAAGCGUAGUGAGUGCGUAAGG
